# A Digital Personal Health Library for Enabling Precision Health Promotion to Prevent Human Papilloma Virus-Associated Cancers

**DOI:** 10.3389/fdgth.2021.683161

**Published:** 2021-07-21

**Authors:** Olufunto A. Olusanya, Nariman Ammar, Robert L. Davis, Robert A. Bednarczyk, Arash Shaban-Nejad

**Affiliations:** ^1^UTHSC-Oak Ridge National Laboratory (ORNL) Center for Biomedical Informatics, Department of Pediatrics, College of Medicine, The University of Tennessee Health Science Center, Memphis, TN, United States; ^2^Hubert Department of Global Health, Rollins School of Public Health, Emory University, Atlanta, GA, United States

**Keywords:** precision public health, health promotion, human papillomavirus, vaccine, screening, cancer prevention, personal health library

## Abstract

Human papillomavirus (HPV) causes the most prevalent sexually transmitted infection (STI) in the United States. Sexually active young adults are susceptible to HPV, accounting for approximately 50% of new STIs. Oncogenic HPV subtypes 16 and 18 are associated with squamous intraepithelial lesions and cancers and are mostly preventable through prophylactic HPV vaccination. Accordingly, this study's objectives are to (1) summarize SDoH barriers and implication for low HPV vaccination rates among young adults (18–26 years), (2) propose a digital health solution that utilizes the PHL to collect, integrate, and manage personalized sexual and health information, and (3) describe the features of the PHL-based app. Through the application of novel techniques from artificial intelligence, specifically knowledge representation, semantic web, and natural language processing, this proposed PHL-based application will compile clinical, biomedical, and SDoH data from multi-dimensional sources. Therefore, this application will provide digital health interventions that are customized to individuals' specific needs and capacities. The PHL-based application could promote management and usage of personalized digital health information to facilitate precision health promotion thereby, informing health decision-making regarding HPV vaccinations, routine HPV/STI testing, cancer screenings, vaccine safety/efficacy/side effects, and safe sexual practices. In addition to detecting vaccine hesitancy, disparities and perceived barriers, this application could address participants' specific needs/challenges with navigating health literacy, technical skills, peer influence, education, language, cultural and spiritual beliefs. Precision health promotion focused on improving knowledge acquisition and information-seeking behaviors, promoting safe sexual practices, increasing HPV vaccinations, and facilitating cancer screenings could be effective in preventing HPV-associated cancers.

## Introduction

Human papillomavirus (HPV) causes the most prevalent sexually transmitted infection (STI) in the United States (1). Each year, the Centers for Disease Control and Prevention (CDC) reports an estimated prevalence and incidence of 42.5 million and 13 million HPV infections, respectively (2). Likewise, STIs are on the rise with approximately 20 million incident cases recorded each year (3, 4). Almost half of all new STIs diagnosed annually affect young adults (15–24 years), who are disproportionately impacted by HPV and other STIs due to risky behaviors such as unprotected sexual intercourse, multiple sexual partners, and reluctance to utilize sexual health services (5, 6). Accordingly, about one in every four sexually active young females become infected with an STI, primarily HPV and chlamydia (5), while approximately half (46.5%) of all young males (23–27 years) report being infected with HPV (7). In 2018, the direct healthcare cost attributed to new STIs had far-reaching financial implications, being estimated at $16 billion (2).

While most HPV infections are asymptomatic or transient due to being cleared by the immune system, the persistence of oncogenic HPV subtypes 16 and 18 is associated with cervical, vaginal, vulvar, anal, penile, and oropharyngeal cancers (8, 9). In the U.S., over 35,900 incident cases of HPV-associated cancers are recorded annually: cervical, 12,143; oropharyngeal, 19,775; anal, 7,083; and vulvar, 4,114 (10). The available HPV vaccines are recognized as primary prevention tools that are effective and protective against approximately 92% of HPV-associated cancers and genital warts (11). Consequently, the Advisory Committee on Immunization Practices recommends that a 9-valent HPV vaccine effective against 9 high- and low-risk HPV subtypes be administered in a two-dose vaccination schedule to females and males 11–12 years old (11). For those without prior vaccination, the Advisory Committee endorses catch-up HPV vaccinations for females and males aged through 26 years (12, 13). Aside from being more susceptible to HPV and STIs, college-age adults (18–26 years) should be targeted for catch-up HPV vaccination interventions because at this age they are making their own health decisions independently of their parents.

Previously, we proposed to develop the Personal Health Library (PHL) that will support chronic disease self-management (14, 15). The PHL is also able to combine and utilize clinical, biomedical, reproductive and sexual health, and social determinants of health (SDoH) data from multi-dimensional sources. These sources include needs assessment surveys, the U.S. Census Bureau American Community Survey, electronic medical records (EMR), wearable/mobile devices, the websites of governmental and public health agencies such as the CDC and World Health Organization (WHO), scientific literature, and data collected via social media platforms. Overall, the PHL can facilitate precision health promotion for HPV vaccine uptake, empower individuals to seek health information, and enable better health decision-making.

The objectives of this article are to (i) summarize SDoH barriers and implication for low HPV vaccination rates among susceptible young adults (18–26 years); (ii) propose a digital health solution that utilizes the PHL to collect, integrate, and manage personalized sexual and health information; and (iii) describe case scenarios and features of the PHL-based app based on ease of use, clinical content, and requirement gathering. The rest of the paper is organized as follows: section 2 describes the role of SDoH barriers in decreasing vaccination rates, section 3 elucidates on health information-seeking and decision-making behaviors, section 4 depicts the application of digital PHL to facilitate precision health promotion, section 5 describes the integration of multi-dimensional data sources to implement the PHL while section 6 characterizes the structure and features of the proposed PHL.

## Role of SDOH Barriers in Decreasing Vaccination Rates Among Susceptible Young Adults

The 2017 Behavioral Risk Factor Surveillance System survey reports that the number of young adults 18–26 years who initiated (21%) or completed (18%) the HPV vaccination series was very low, particularly when compared with the Healthy People 2030 target of 80% for HPV vaccine uptake by ages 13–15 years (16). Therefore, within a college/university setting, it is likely that a significant number of sexually active young adults are unvaccinated. Given their potentially greater propensity for unsafe, at-risk behavioral practices compared with other age groups, these young adults are vulnerable to HPV infections and therefore susceptible to HPV-associated cancers. The 2017 survey also indicates that 18–26 year-old men were least likely to be vaccinated (16). It is of concern that men who have sex with men or identify as gay or bisexual have an increased prevalence of HPV infection (17).

**Figure 1:**
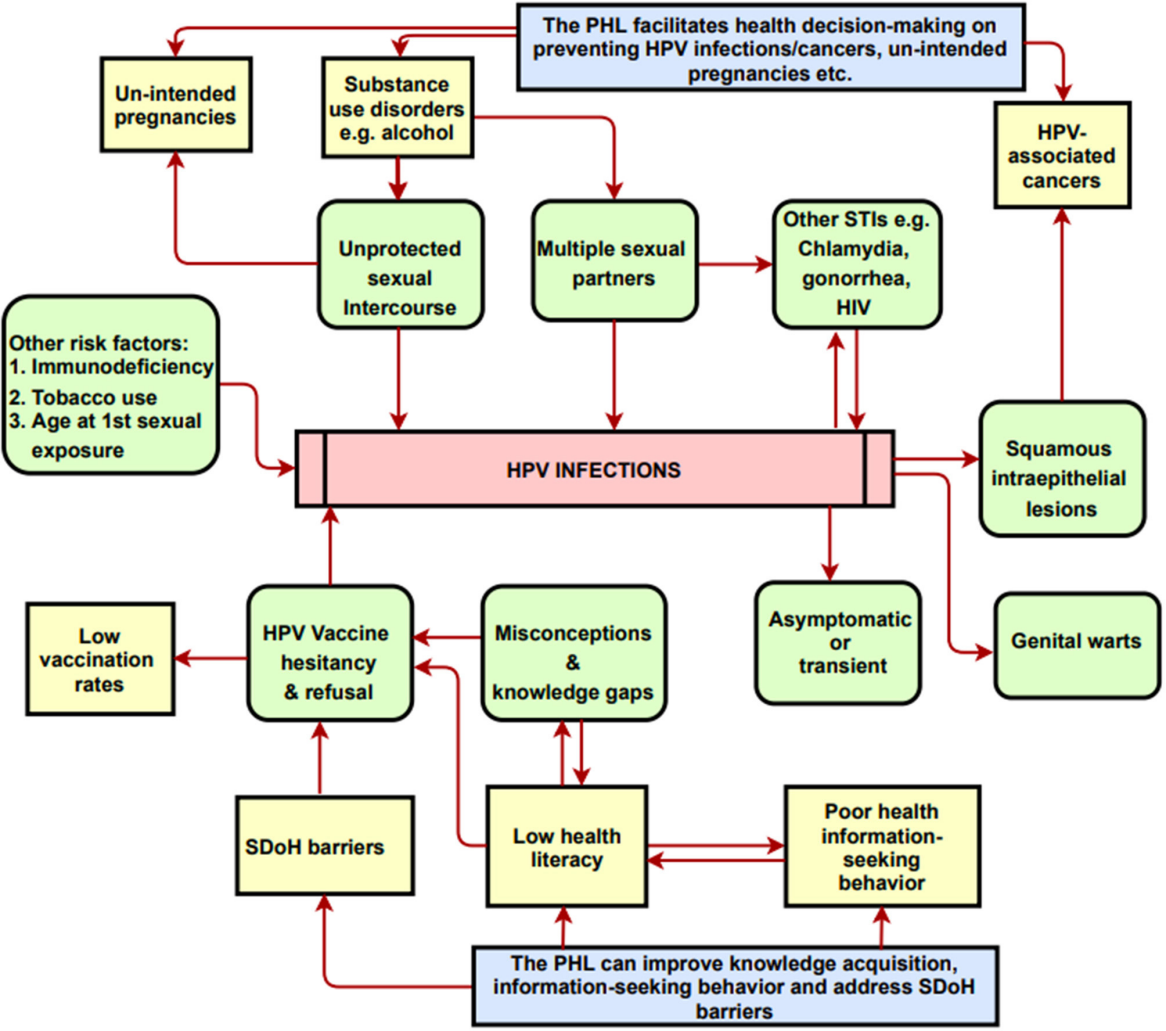
A conceptual model on the potential impacts of the PHL-based app on knowledge and health decision-making. PHL, Personal Health Library; SDoH, Social Determinants of Health. The Conceptual Model Diagram depicts the risk factors and/or correlates for HPV infections and cancers. Arrows represent partial correlation/partial causality between the entities shown in the diagram. Diagram also shows how the PHL addresses risks factors and SDoH barriers.

Sociocontextual determinants can hinder HPV vaccine uptake behavior. SDoH embodies the characteristics of neighborhoods, communities, and the environments in which individuals are born, reside, learn, work, and worship. Specifically, SDoH are influenced by the availability of resources that improve quality of life and public health outcomes, including income, access to education, affordable housing and basic amenities, health services, public safety, and food security (18). Among young adults, SDoH barriers adversely influence HPV vaccine acceptance and ultimately lead to vaccine delay, hesitancy, and refusal (19). These barriers include lack of health-related knowledge, low health literacy regarding vaccine safety/efficacy/side-effects, disparities in health information-seeking behaviors, out-of-pocket vaccine cost, poor healthcare access, inadequate or absent health insurance, limited access to healthcare providers' recommendations, language barriers, sub-optimal digital literacy level, peer influence, and parental religious and moral viewpoints (Table 1 and Figure 1).

**Table 1:** Barriers to vaccine uptake based on the CDC's classification of the SDoH domains.

**SDoH domains**	**Contents**
Education	Educational attainment, health-related knowledge, health literacy, health information-seeking behavior, language development, technical skill.
Health and healthcare	Healthcare access, health insurance coverage, health literacy, proximity to healthcare facility, availability of healthcare provider.
Economic stability	Poverty, employment, safe and affordable housing, out-of-pocket vaccine cost, internet access.
Neighborhood and built environment	Environmental conditions, access to transportation, proximity to a healthcare facility, average household size, neighborhood crime and violence.
Social and community context	Health providers' recommendations, patient-provider communication, peer influence, parental religious and moral viewpoints, stressful life event impacting the family/household, incarceration.

## Health Information-Seeking and Decision-Making in the Digital Age

Several studies have shown that access to education and to clear, unequivocal recommendations/policies serve as predictors for increasing vaccine uptake (20). However, young adults report a lack of knowledge and awareness of HPV, its transmissibility through skin-to-skin contact, the existence of HPV-associated cancers, and HPV vaccine efficacy and protocols (21–23). Additionally, most college-age students did not perceive themselves to be at risk of acquiring HPV (24). In today's internet era, people are increasingly knowledgeable about navigating digital and mobile devices, online social media platforms to seek and control their own health information, given the proper motivation to do so (25). Moreover, several studies have demonstrated that digital health technologies can increase knowledge, inform health decision-making and change HPV vaccination behaviors (26, 27). Therefore, digital health technologies are uniquely and ideally suited to disseminating information and to facilitating social support that could increase vaccine uptake. As depicted in Figure 1, digital health technologies and applications could serve as resource hubs for promoting HPV education, awareness, vaccination services, cancer prevention measures, and for addressing vaccine hesitancy and misinformation, thereby ultimately reducing the incidence of HPV-associated cancers.

## The Application of Digital PHL to Facilitate Precision Health Promotion

Precision health promotion, one of the main pillars of digital precision health (28), is defined as, “the personalized design of lived experiences that foster improved health and well-being for individuals within the context of families, organizations and communities” (29). Therefore, an education-focused intervention with strong, tailored, and consistent health messaging communicated through a digital health application could play an important role in facilitating the voluntary behavior of getting vaccinated against HPV. The PHL is a digital tool that could facilitate informed health decision-making and lifestyle choices through customized health information that is collected, integrated, organized, managed, and retrieved through the application of novel techniques. Technologies such as artificial intelligence, knowledge representation, semantic web capabilities, and natural language processing could be used to design the PHL resource.

**Figure 2:**
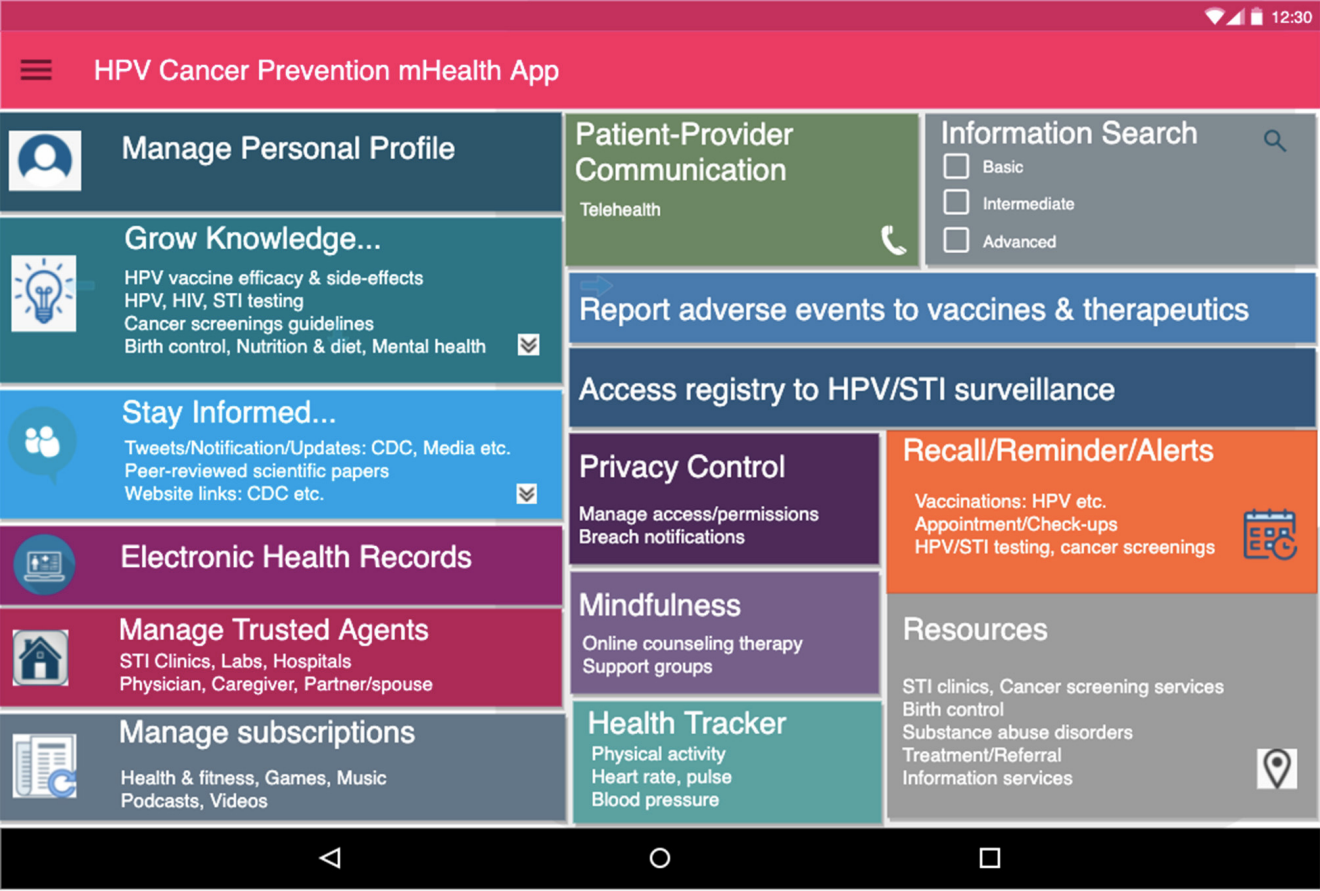
Proposed dashboard interface for PHL-based application. Initial user-friendly interface for PHL-based app. The app supports the following: (a) Social features (b) utilizes SDoH data e.g. access to nearby resources (c) facilitates knowledge acquisition and recommendations on safe sex behavior (e) manages trusted agents (including partners, physicians, family members) and subscriptions.

The proposed PHL-based application has the potential to leverage the following capabilities: (i) integrating individual- and population-level data and evidence; (ii) custom-building to each individual's specific needs with a patient-centered design; (iii) enabling knowledge acquisition, exchange, validation, and visualization; (iv) promoting healthy behaviors and improving health-information-seeking behaviors; (v) utilizing patient reminder and recall systems; and (vi) learning usage patterns to incorporate an individual's spiritual, moral and cultural preferences. As shown in Figures 1, 2, the application provides intelligent, personalized health education and promotion that address knowledge gaps and misconceptions regarding sexual health; the safety, efficacy, side effects, and cost associated with HPV vaccines; and other cancer-preventive measures such as routine testing for HPV and other STIs and Pap testing to screen for atypical cell morphologies in cervical and other tissues. Overall, this could serve as an individual-centered digital tool tailored for tracking and exchanging information to inform health decisions regarding HPV, STIs, and cancer prevention. It can also be adapted to include other health-related issues among adults of all ages such as substance use and mental health disorders.

## Integrating Data From Multi-Dimensional Sources for the PHL

To implement the proposed PHL, complex multi-dimensional health information (e.g., clinical, biomedical, and SDoH characteristics) is compiled and integrated from multiple heterogeneous sources at individual- and population levels and from trusted domains. Below, we discuss the PHL sources and types of data and information that are included in further detail.

### Individual-Level Data

#### Electronic Health Records

Electronic health records (EHRs) systematically collect patients' data that are captured in a clinical setting and stores it in a digital format. EHRs capture a wide range of information including health histories, prescriptions, biomedical and laboratory results, immunizations, demographics including age and home address, and information about disease progression and medical treatments. This data can be complex and multilayered, so the application of artificial intelligence techniques to identify patterns and generate organized data for analysis could facilitate greater insight to inform individual's health decision-making.

#### Patient-Generated Health Data and Observations of Daily Living

Patient-Generated Health Data (PGHD) and Observations of Daily Living (ODL) are generated from consumer-health apps, sensors, and wearable devices. Compared with clinical EHR data, the PGHD records of health-related data (e.g., glucose and cholesterol monitoring, medical history, feedback on medication, and adverse drug reports) are gathered, distributed, and controlled by the patients themselves. Studies have revealed that these wearable devices are relatively accurate for remotely monitoring, tracking and measuring physiological metrics (e.g., heart rate, temperature, blood saturation level) and behavior (e.g., physical activity, sleep) (30). ODLs incorporate data on the overall well-being, health, and fitness of an individual, including physical activity, heart rate and pulse. Records from PGHD and ODL can be used to enable and promote healthy behaviors by generating “personalized” information and data.

### Population-Level Data

#### SDoH Characteristics

The adoption of SDoH factors to facilitate catch-up HPV vaccinations represents a novel approach. SDoH data and neighborhood characteristics are obtained and curated at the zip code/census tract level from the Census Bureau and local partners. The Census Bureau utilizes the American Community Survey as its source of information to depict changing U.S. demographics (e.g., housing, average household size, employment, and internet access). This population-level data are integrated with individual-level characteristics to design the PHL-based application.

#### Needs Assessment Survey

The primary target population for this survey is a diverse, representative sample of young adults (18–26 years) affiliated with U.S. universities and colleges. The recruitment will be conducted via groups on Facebook (and potentially other social media platforms as needed) and will incorporate invitations for participation that include web link access to the needs assessment survey. A structured online questionnaire will be administered to determine individuals' specific needs for a digital application within a socio-cultural context including multilingualism, home access, internet connectivity, and digital tool proficiency. Data will also be collected about knowledge and attitudes regarding HPV, HPV vaccination and vaccine hesitancy. Together, these results will inform the design of the PHL-based application.

### Other Trusted Sources of Data and Resources

#### Public Health Agency Websites and Peer-Reviewed Scientific Literature

To enhance the usability of the PHL platform, science-based evidence and data from public health agency websites (governmental such as CDC and non-governmental) and scientific literature from peer-reviewed journals will be incorporated. The CDC, the U.S. National Cancer Institute, and WHO serve as research data archives that enable public access to information on reproductive and sexual health, HPV and STIs, vaccinations, screenings, cancer prevention, and surveillance. Additionally, the Substance Abuse and Mental Health Services Administration offers confidential free information services for treatment and referrals to address mental health crises, substance-use disorders, and other behavioral health disorders.

#### Social Media, Web Blogs, and Podcasts

Behavioral health data, spiritual and cultural viewpoints, general beliefs, and opinions presented in formats such as posts, messages, blogs, podcasts, and video recordings on online social media all serve as valuable sources of information for the PHL.

## The Structure and Features of the Proposed PHL-Based Application

We would create an integrated dynamic knowledge base using these multiple data sources to enable PHL to generate real-time hybrid recommendations that are based on both context and content. To capture context, the PHL integrates individual-level ODL and PGHD data, and population-level data such as SDoH and neighborhood characteristics. The PHL subsequently transforms this information into a machine-readable format using semantic web technologies and natural language processing. This in turn facilitates interoperability between the PHL and other platforms via ontologies and semantic networks of knowledge types that are related to HPV and STIs prevention and cancer screenings, our domain of interest. The technical details underlying the PHL infrastructure have been described and previously published (14, 15, 31).

### Proposed PHL-Based Application in Action: Case Scenarios

Here, we present sample scenarios to demonstrate the features, ease of use, clinical content, and requirement gathering already implemented in the initial prototype design of the PHL-based health promotion app. It is important to note that the case scenarios and user features illustrated below should be supported and justified by empirical data from our future work.

- **Scenario 1**: A 19-year-old college sorority female member wants to use the app to acquire knowledge so she can establish appropriate norms to discourage sexual risk during a party she plans to organize.
- **Scenario 2**: A 22-year-old male who newly identifies as gay distrusts the healthcare system and fears discrimination because of his lifestyle choices. He is a recent immigrant with little English proficiency. He seeks social support and information on the PHL-based app to learn how he and his new partner can engage in safe sex practices within their relationship, thereby preventing HPV and STIs.
- **Scenario 3**: A 20-year-old male college athlete who has not been vaccinated for HPV has poor health-seeking behavior. However, he feels he is invincible and immune to getting HPV and STIs despite having multiple sexual partners and engaging in unprotected sexual intercourse. His friend suggests that he sign up to receive daily messages on his phone through the PHL-based app for information about HPV and STIs prevention measures including condom use, vaccinations, and STI testing.
- **Scenario 4**: A 19-year-old female undergraduate student engaged in unprotected sexual intercourse the previous night. She fears she may have contracted HPV or an STI and seeks privacy as she remotely consults a nurse using the PHL-based app. She also utilizes the app's sexual health information to inform her decisions about HPV and STI testing, catch-up HPV vaccinations, and emergency birth control measures.

#### PHL-Based Application Features and Thematic Assessment of Requirements

The PHL-based app requirements for these case scenarios are listed below, derived mostly from HPV-associated cancer preventive measures, i.e., safe sexual practices, HPV vaccine uptake, routine HPV testing, and cancer screenings. Each requirement is also mapped to features that would be part of the proposed PHL-based app, as seen in Figure 2.

##### Scenario 1

- **PHL-based app features**: (i) social features: chatting channels, blogs, applications, and podcasts, (ii) web links to CDC, etc., and (iii) recommendations on safe sexual behaviors and safer sex guidelines.
- **Requirements addressed:** (i) safe-sex practices such as condom use, (ii) avoidance of alcohol and drugs, (iii) routine HPV/HIV/STI testing, and (iv) peer support.

##### Scenario 2

- **PHL-based app features:** (i) social features: chatting channels and shared notepads, (ii) SDoH characteristics: access to nearby sexual health resources and information, (iii) management of trusted sex partners, (iv) recommendations on safe-sex behavior and safer-sex guidelines, (v) recalls and reminders for vaccinations, STI tests, and cancer screenings, (vi) privacy and confidentiality, and (vii) language preferences.
- **Requirements addressed:** (i) Safe sexual practices including condom use, (ii) HPV vaccine uptake, (iii) routine HPV/HIV/STI testing, (iv) cancer screenings, and (v) privacy and confidentiality.

##### Scenario 3

- **PHL-based app features**: (i) knowledge acquisition, (ii) facilitation in seeking health information, (iii) integration with global web knowledge, (iv) recommendations on safe sex behavior and safer sex guidelines, and (v) incorporation of spiritual and cultural viewpoints.
- **Requirements addressed:** (i) safe sex practices including condom use, (ii) HPV vaccine uptake, (iii) vaccine hesitance, (iv) routine HPV/HIV/STI testing, and (v) peer support.

##### Scenario 4

- **PHL-based app features**: Social features: chatting channels, shared notepads, (ii) video conference and telehealth, (iii) recommendations on safe sex behavior, (iv) reminders for cancer screenings and testing, (v) manages trusted sex partners, thereby facilitating contact tracing, and (vi) privacy and confidentiality.
- **Requirements addressed:** (i) Safe sex practices including condom use, (ii) HPV vaccine uptake, (iii) routine HPV/HIV/STI testing, (iv) birth control options, (v) privacy and confidentiality, and (vi) contact tracing.

## Conclusions and Future Directions

The immune response elicited by HPV vaccines is effective in preventing infections from the high-risk HPV subtypes (16 and 18) responsible for 92% of HPV-associated cancers. Primary prevention including HPV vaccine uptake and use of safe sex practices, secondary prevention, early detection through HPV testing and cancer screenings, and lifestyle changes such as smoking cessation and reduced consumption of alcohol could prevent approximately 400,000 HPV-associated cancers annually (32). Overall, we aim to increase catch-up HPV vaccinations and address vaccine hesitancy through precision health promotion, thus ultimately reducing the prevalence of HPV-associated cancers.

In this article, we discuss the utility of personalized digital health solutions for facilitating and promoting healthy behaviors focused on HPV vaccine uptake and cancer screenings. We also propose to continue our research on the design and development of an intelligent PHL-based application that improves health-seeking behavior and decision-making by disseminating personalized recommendations through a user-friendly interface. We will use the results from the needs assessment survey to implement, optimize, and tune the PHL-based app, which will facilitate the gathering, managing, and use of personalized health information. Moreover, we define a series of case scenarios to highlight the features and user requirements implemented in the app.

Future work will entail the completion and incorporation of a formal needs assessment survey results into developing the PHL-based app as well as the evaluation of the PHL prototype by a panel of subject matter experts for clarity, ease of use, workflow, content, and omission. The evaluation will be conducted in the form of (i) semi-structured interviews to obtain qualitative data and (ii) review of case scenarios to simulate the cognitive process of individuals interacting with the app and attempting to make health decisions. Moreover, when fully implemented, a formal usability assessment with a specific focus group will be performed. Future studies should expatiate on the potential impact of the proposed app in quantitative measures, (e.g., what is the impact of the app on HPV vaccination rates and other behavior change outcomes, what is the satisfaction rate for the app?) among different demographic groups and varying disease conditions. Anticipated limitations to fully implementing the PHL-based app include concerns and challenges about data privacy and data breach. Concerted efforts should be made to enforce privacy-preserving mechanisms at multiple levels (33) as well as to utilize anonymized data from willing participants only.

A comprehensive novel intervention that is education-focused and employs a digital health application to collect, control, and utilize contextualized personal data and information would be empowering and impactful for promoting HPV-associated cancer preventive measures. Importantly, this intervention will be vital in reducing the burden of HPV-associated diseases among young adults.

## Data Availability Statement

The original contributions presented in the study are included in the article/supplementary material, further inquiries can be directed to the corresponding authors.

## Author Contributions

OO: conceptualization, writing the draft, review, and editing, visualization. NA: conceptualization, review and editing. RD and RB: review and editing. AS-N: conceptualization, writing, review and editing, obtained funding, supervision. All authors contributed to the article and approved the submitted version.

## Conflict of Interest

The authors declare that the research was conducted in the absence of any commercial or financial relationships that could be construed as a potential conflict of interest.
